# Establishment of a male fertility prediction model with sperm RNA markers in pigs as a translational animal model

**DOI:** 10.1186/s40104-022-00729-9

**Published:** 2022-07-07

**Authors:** Won-Ki Pang, Shehreen Amjad, Do-Yeal Ryu, Elikanah Olusayo Adegoke, Md Saidur Rahman, Yoo-Jin Park, Myung-Geol Pang

**Affiliations:** Department of Animal Science & Technology and BET Research Institute, Chung-Ang University, Anseong, Gyeonggi-do 17546 Republic of Korea

**Keywords:** Male fertility, Multiple markers, Pig model, Prediction model, Sperm RNA

## Abstract

**Background:**

Male infertility is an important issue that causes low production in the animal industry. To solve the male fertility crisis in the animal industry, the prediction of sperm quality is the most important step. Sperm RNA is the potential marker for male fertility prediction. We hypothesized that the expression of functional genes related to fertilization will be the best target for male fertility prediction markers. To investigate optimum male fertility prediction marker, we compared target genes expression level and a wide range of field data acquired from artificial insemination of boar semen.

**Results:**

Among the genes related to acrosomal vesicle exocytosis and sperm–oocyte fusion, equatorin (*EQTN*), zona pellucida sperm-binding protein 4 (*ZP4*), and sperm acrosome membrane-associated protein 3 exhibited high accuracy (70%, 90%, and 70%, respectively) as markers to evaluate male fertility. Combinations of *EQTN*-*ZP4*, *ZP4*-protein unc-13 homolog B, and *ZP4*-regulating synaptic membrane exocytosis protein 1 (*RIMS1*) showed the highest prediction value, and all these markers are involved in the acrosome reaction.

**Conclusion:**

The *EQTN*-*ZP4* model was efficient in clustering the high-fertility group and may be useful for selection of animal that has superior fertility in the livestock industry. Compared to the *EQTN*-*ZP4* model, the *ZP4*-*RIMS1* model was more efficient in clustering the low-fertility group and may be useful in the diagnosis of male infertility in humans and other animals. The appointed translational animal model and established biomarker combination can be widely used in various scientific fields such as biomedical science.

**Supplementary Information:**

The online version contains supplementary material available at 10.1186/s40104-022-00729-9.

## Introduction

Male infertility is a severe problem that extends across all animals. In humans, increasing male infertility threatens the deterioration of next-generation and eventually, the entire human race. The livestock industry comprising cow, pig, and chicken also suffers from male fertility issues. In cows and pigs, male infertility is directly correlated with the economics of the livestock industry and world food production. Concerning this issue, many scientists point out that a decrease in sperm quality causes male infertility. Therefore, the precise assessment of sperm quality is the first step to treat male infertility. Traditionally, sperm quality is evaluated employing laboratory techniques assessing sperm motility and viability [1, 2]. However, the clinical acceptance of these methods is still uncertain in many species [3, 4].

There is an emphasis on the importance of an omics approach to predict male fertility, instead of conventional laboratory assays [5–7]. Many studies indicate the potential of sperm RNA as a diagnostic tool for assessing male infertility [8–10]. Spermatozoa contain mRNAs, fragmented tRNAs, tsRNAs, miRNAs, lncRNAs, piRNAs, and siRNAs [11, 12]. Sperm RNAs are delivered to the oocyte [13] and serve critical roles like transportation of paternal phenotype [14–17], epigenetic modification of offspring genome [18], and aid the detection of transcripts to guide normal embryogenesis and implantation [19]. Moreover, comprehensive omics studies have revealed that the differential expression of sperm RNA is related to different fertility statuses in humans, porcine, bovine, ovine, and equine species [20–24]. Despite the functional and clinical importance of sperm RNA, studies that use RNA to evaluate male fertility are still in the experimental stage due to the lack of an accurate fertility model for clinical trials.

Pig, as an animal model, contributes greatly to biomedical research, particularly studies on obesity, arthritis, cardiovascular disease, skin and eye conditions, and xenotransplantation [25, 26]. Compared to rodent models, pigs are more physiologically related to humans [27]. In addition, pig fertility is digitized accurately through artificial insemination (AI) results [28]. Considering such diverse characteristics, pigs are an adequate animal model to study the clinical acceptance of sperm RNA on male fertility in animals, including humans.

The sperm-oocyte interaction is the starting point of embryonic development, and for successful interaction, all molecular mechanisms must work in harmony [29]. Before sperm-oocyte interaction, the acrosome of the sperm undergoes several changes for fusion with the zona pellucida. This biological process, called the acrosome reaction, involves acrosomal exocytosis, substructure remodeling, and other biochemical modifications [30]. These processes are inevitable for the successful fusion of sperm to the oocyte plasma membrane in single fertilization [31]. The normal functioning of genes related to the aforementioned mechanisms leads to fertility success, and the expression pattern of genes is closely related to the corresponding mechanisms. While such genes provide early clues regarding male fertility in a rodent model [13], little is known about their function in humans and other large animals. Considering their important roles in rodents, we postulated that they may play a key role in male fertility in other large animals. Therefore, in this study, we aimed to identify male fertility markers by screening the mRNA expression levels of genes that encode proteins crucial for acrosomal vesicle exocytosis and sperm–oocyte fusion in pig spermatozoa. To understand the physiological roles of these genes in male fertility in other large animals, we selected pig as the translational animal model of male fertility. We also concluded the accuracy and sensitivity of our protocol by employing large samples of boar semen representing a wide range of levels of field data.

## Materials and methods

### Experimental design

Firstly, ‘acrosomal vesicle exocytosis’ and ‘sperm-egg fusion’ related genes were listed to investigate relevant boar fertility prediction marker models.

Secondly, to narrow down target genes, high (average litter size = 13.97 ± 0.07)- and low (average litter size = 11.17 ± 0.13)-fertility representative samples (*n* = 3) were chosen based on total litter size data after AI trials. The differentially expressed genes in high- and low-fertility groups were progressed to further process.

Thirdly, the expression of genes from the second step was tested in 20 randomly selected Yorkshire boars and then compared with litter size. The genes that were significantly correlated with litter size were subjected to marker evaluation with the receiver operating characteristic (ROC) curve analysis.

Finally, each evaluated markers were combined to improve boar fertility prediction value. Multiple marker model predictability of litter size (over and under cut-off value) was evaluated with ROC curve analysis. Multiple marker models which exhibited outstanding prediction value in the previous analysis were subjected to further statistical analysis. The predictability of marker models to distinguish boars into three groups (high, medium, and low) was evaluated to develop an optimum combination.

### Animal care

All procedures involving animals were approved by the Institutional Animal Care and Use Committee of Chung-Ang University (approval number: 2017–00018). All boars and sows used in the experiment were reared at 20 ± 5 °C under a 2:1 light/dark cycle, continuous air circulation, and appropriate feed and water.

### Sperm sample preparation

A total of 26 Yorkshire boar (body weight over 90 kg and age between 9 to 24 months) semen was collected using the gloved-hand technique [32] from Sunjin Co. (Danyang, Korea). The motile spermatozoa were separated from semen with discontinuous 35% and 70% Percoll gradient (Sigma-Aldrich Co., St. Louis, MO, USA) [33]. The spermatozoa were cultured using modified tissue culture media 199 (containing 10% fetal bovine serum [v/v], 0.91 mmol/L sodium pyruvate, 3.05 mmol/L D-glucose, 2.92 mmol/L calcium lactate, and 2.2 g/L sodium bicarbonate) (Sigma-Aldrich Co.) under 5% CO_2_ and at 37 °C for 30 min [34]. The sperm samples were pelleted and then deep-frozen with liquid nitrogen (− 196 °C) and stored at − 80 °C before use.

### Sperm motility and motion kinematics assessment

Sperm motility and motion kinematics were analyzed by a computer-assisted sperm analysis system (SAIS-Plus ver.10.1; Medical Supply, Seoul, Korea) [35]. Washed spermatozoa were resuspended in modified tissue culture media 199. Then, under a microscope, the motility (%), hyperactivation (HYP; %), curvilinear velocity (VCL; μm/s), straight-line velocity (VSL; μm/s), average path velocity (VAP; μm/s), linearity (LIN; %), beat cross frequency (BCF; Hz), wobble (WOB; %), and mean amplitude of head lateral displacement (ALH; %) of the spermatozoa were measured on a pre-heated (37 °C) Makler counting chamber (Sefi Medical Instruments, Haifa, Israel). For a single analysis, 200–300 spermatozoa were subjected, and all samples were analyzed three times.

### Capacitation status assessment

The spermatozoa were stained with chlortetracycline (Sigma-Aldrich Co.) and Hoechst 33258 (Sigma-Aldrich Co.) and their capacitation status were measured in approximately 400 spermatozoa per slide [36, 37]. Each sample was smeared into three slides. Briefly, the incubated spermatozoa are stained with Hoechst 33258 solution. Then, the excess dye was inactivated with a 2% polyvinylpyrrolidone (Sigma-Aldrich) solution. Stained spermatozoa were pelleted and then resuspended in 600 μL of PBS and 600 μL of chlortetracycline (CTC) fluorescence solution (750 mmol/L CTC in 5 μL buffer; 20 mmol/L Tris, 130 mmol/L sodium chloride, and 5 mmol/L cysteine, pH 7.4) (Sigma-Aldrich). The stained samples were counted under epifluorescence illumination with UV BP 340–380/LP 425 and BP 450–490/LP 515 excitation/emission filters for Hoechst 33258 and CTC, respectively, using a Microphot-FXA microscope (Nikon, Tokyo, Japan). Sperm capacitation status was further classified into four categories: live non-capacitated (F; yellow fluorescence distributed evenly throughout the sperm head), live capacitated (B; yellow fluorescence over the acrosome region and a dark post-acrosome region), acrosome-reacted (AR; showing no fluorescence over the head), and dead (D; nuclei with blue fluorescence within the sperm head).

### Sperm RNA isolation

Sperm RNA was isolated according to a previous study [38]. Briefly, sperm numbers were adjusted to 40–50 × 10^6^ cells/mL. Sperm pellets were suspended in non-toxic guanidine-isothiocyanate lysis buffer containing β-mercaptoethanol (40 μL/mL; Sigma-Aldrich Co.) and homogenized using a 20G syringe. TRIzol (500 μL) (Invitrogen, Carlsbad, CA, USA) and chloroform (200 μL) were added to the homogenized sample and centrifuged at 12,000 × *g* for 25 min. After centrifugation, the upper layer (500 μL) was moved to a fresh tube and mixed with pure ethanol (500 μL) (Sigma-Aldrich Co.). Sperm RNA was attached to a spin cartridge and washed with the PureLink RNA Mini Kit (Invitrogen) wash buffer 1 and 2. The isolated RNA was immersed in 20 μL of nuclease-free water (60 °C). The quality (260/280 ratio) and quantity of isolated RNA were measured using an Epoch Microplate Spectrophotometer (BioTek, Winooski, VT, USA).

### Reverse transcription-quantitative real-time polymerase chain reaction

Reverse transcription was performed using PrimeScript 1st strand cDNA Synthesis Kit (Takara Bio, Inc., Shiga, Japan) according to the manufacturer’s protocol. Total 400 ng of sperm RNA from each sample was reverse transcribed and 8–11 μg of cDNAs were yielded. The relative expression of target genes was quantified using the 7500 fast real-time PCR system (Applied Biosystems, Foster City, CA, USA). SYBR Green PCR master mix (Applied Biosystems) was used. The total reaction volume was 20 μL (100 ng of cDNA), and the cycling conditions were set to initial denaturation at 95 °C (10 min) followed by 40 cycles at 95 °C for denaturation (15 s) and 60 °C for annealing (60 s). The results were analyzed using the 7500 Software v2.3 (Thermo Fisher Scientific, Waltham, MA, USA) using the 2^-∆∆Cq^ method. All primers used for qPCR were designed based on Sscrofa11.1 Genome Assembly from The Swine Genome Sequencing Consortium (Additional file 1: Table S1).

### Artificial insemination

Male fertility status of randomly selected 20 Yorkshire boars was evaluated from the results obtained after AI using their semen. Boar semen was diluted with 100 mL of the Beltsville thawing solution (30 × 10^6^ sperm cells/mL) [39]. AI was performed twice per estrus [40] in an average of 19.3 ± 1.2 sows per boar (total 386 trials). The average number of piglets born was considered as the male fertility status of the corresponding boar.

### Bioinformatics

The Gene Ontology database was used to categorize target genes related with the functional classes “acrosomal vesicle exocytosis” and “sperm–egg fusion.” The listed genes were applied to the Pathway Studio program (Elsevier) to visualize their biological functions.

### Statistics

All data obtained were tested for normality and homogeneity of variance. The normality test (Shapiro–Wilk test), homogeneity of variances test (Levene’s test), Student’s *t*-test, linear and multiple regression test, and ROC curve analysis were performed using the SPSS v1.8 software (SPSS, Inc., Chicago, IL, USA). principal component analysis (PCA), *k*-medoids clustering, silhouette plotting, and heatmap analysis were conducted using the R software (RStudio, Boston, MA, USA). *P* < 0.05 was considered to indicate significantly different results and was correlated with fertility. Standard error of the mean was used to show sampling distribution.

## Results

### Sperm motility, motion kinematics, and capacitation status of boar spermatozoa

The functional parameters of spermatozoa, including sperm motility, hypermotility, motion kinetics, and capacitation status were analyzed from all boar spermatozoa. No functional parameter showed a significant difference between the high- and low-fertility groups (Table 1). The sperm motility, motion kinematics, and capacitation status of 20 randomly selected Yorkshire boars were shown in Additional file 1: Table S2.

**Table 1:** Functional parameters of spermatozoa and litter size of the high- and low-fertility groups

	High fertility	Low fertility
Litter size	13.97 ± 0.07*	11.17 ± 0.13
MOT, %	72.94 ± 18.26	70.19 ± 14.68
HYP, %	13.19 ± 6.80	12.67 ± 7.57
VCL, μm/s	134.59 ± 21.57	134.21 ± 25.00
VSL, μm/s	58.66 ± 9.32	65.51 ± 16.53
VAP, μm/s	69.96 ± 11.02	74.29 ± 14.75
LIN, %	43.87 ± 3.14	49.25 ± 7.96
BCF, Hz	13.75 ± 1.10	14.09 ± 1.28
WOB, %	52.10 ± 1.90	55.94 ± 5.31
ALH, μm	5.96 ± 0.92	6.01 ± 1.01
AR, %	2.68 ± 0.88	8.20 ± 6.95
F, %	65.24 ± 10.42	76.92 ± 9.32
B, %	32.08 ± 10.58	14.88 ± 2.80

*MOT* motility, *HYP* hyperactivation, *VCL* curvilinear velocity, *VSL* straight line velocity, *VAP* average path velocity, *LIN* linearity, *BCF* beat cross frequency, *WOB* wobble, *ALH* mean amplitude of head lateral displacement, *AR* acrosome reacted, *F* normal, *B* capacitated. Data are expressed as mean ± SEM; *P < 0.05

### Expression of fertility-related genes in the high- and low-fertility groups

**Fig. 1:**
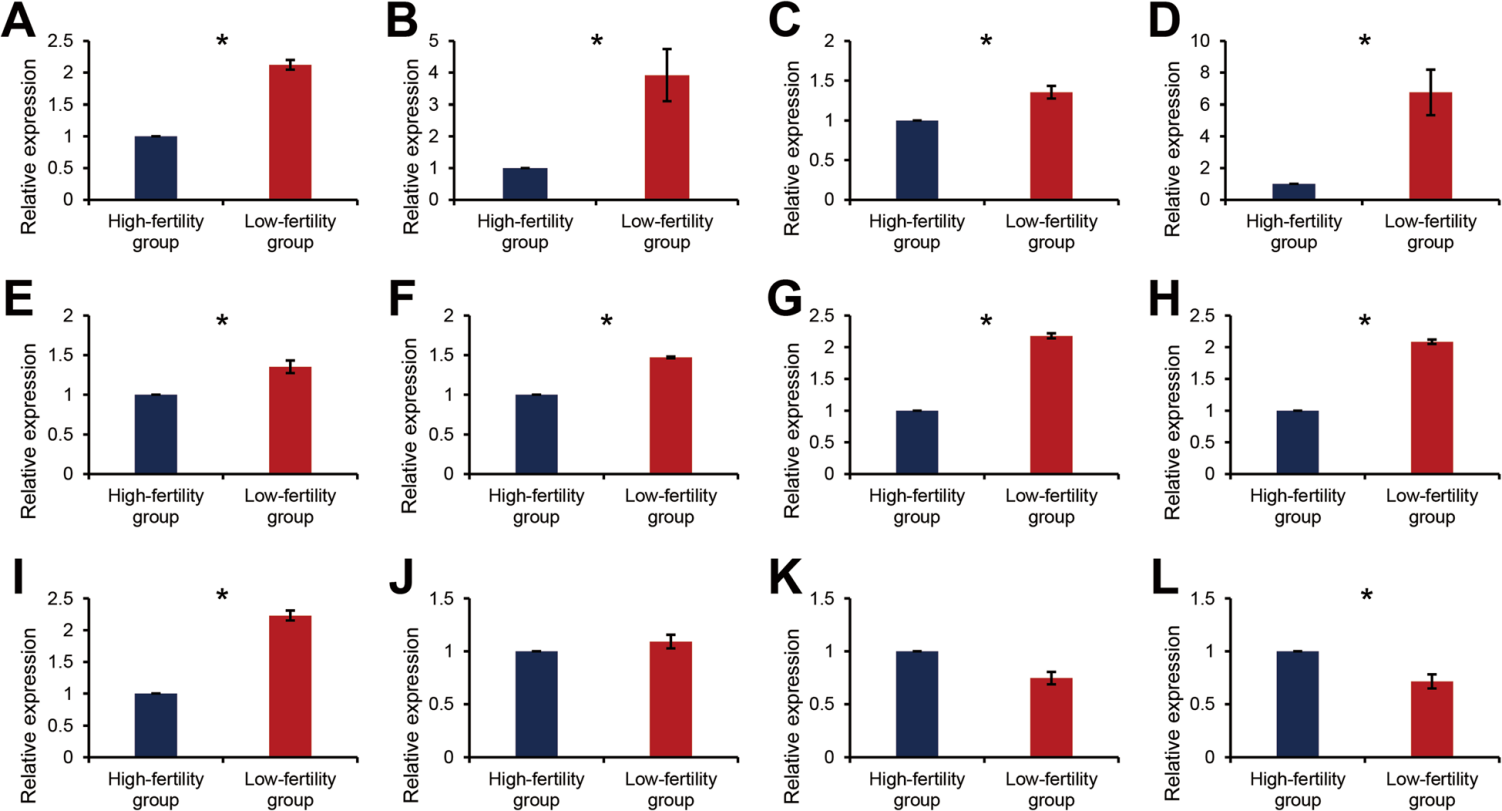
The expression pattern of target genes in the high- and low-fertility groups. Relative mRNA expression of (**A**) *ZP4*, (**B**) *RIMS1*, (**C**) *RAB3A*, (**D**) *UNC13B*, (**E**) *SYT6*, (**F**) *EQTN*, (**G**) *IZUMO1*, (**H**) *SPACA3*, (**I**) *CD9*, (**J**) *LYZL6*, (**K**) *SPATA46*, and (**L**) *IZUMO1R* in the high- and low-fertility groups. (*n* = 3). * *P* < 0.05

We found that equatorin (*EQTN*), zona pellucida sperm-binding protein 4 (*ZP4*), regulating synaptic membrane exocytosis protein 1 (*RIMS1*), Ras-related protein Rab-3A (*RAB3A*), protein unc-13 homolog B (*UNC13B*), and synaptotagmin-6 (*SYT6*) are involved in acrosomal vesicle exocytosis. Moreover, *EQTN*, Izumo sperm–egg fusion protein 1 (*IZUMO1*), sperm acrosome membrane-associated protein 3 (*SPACA3*), *CD9*, lysozyme like protein 6 (*LYZL6*), spermatogenesis-associated protein 46 (*SPATA46*), and IZUMO1 receptor (*IZUMO1R*) are involved in sperm-oocyte fusion. All genes associated with acrosomal vesicle exocytosis are differentially expressed in the high and low litter size groups (Fig. 1A–E; *P* < 0.05). Of the genes categorized in “sperm–egg fusion” *EQTN*, *IZUMO1*, *SPACA3*, *CD9*, and *IZUMO1R* were differentially expressed in the high and low litter size groups (Fig. 1E–I and L; *P* < 0.05). *LYZL6* and *SPATA46* were not differentially expressed according to litter size (Fig. 1J and K, *P* < 0.05). Of the twelve genes investigated, ten differentially expressed genes were selected to be further evaluated as candidates for male fertility markers in validation trials.

### Assessment of single fertility marker

**Fig. 2:**
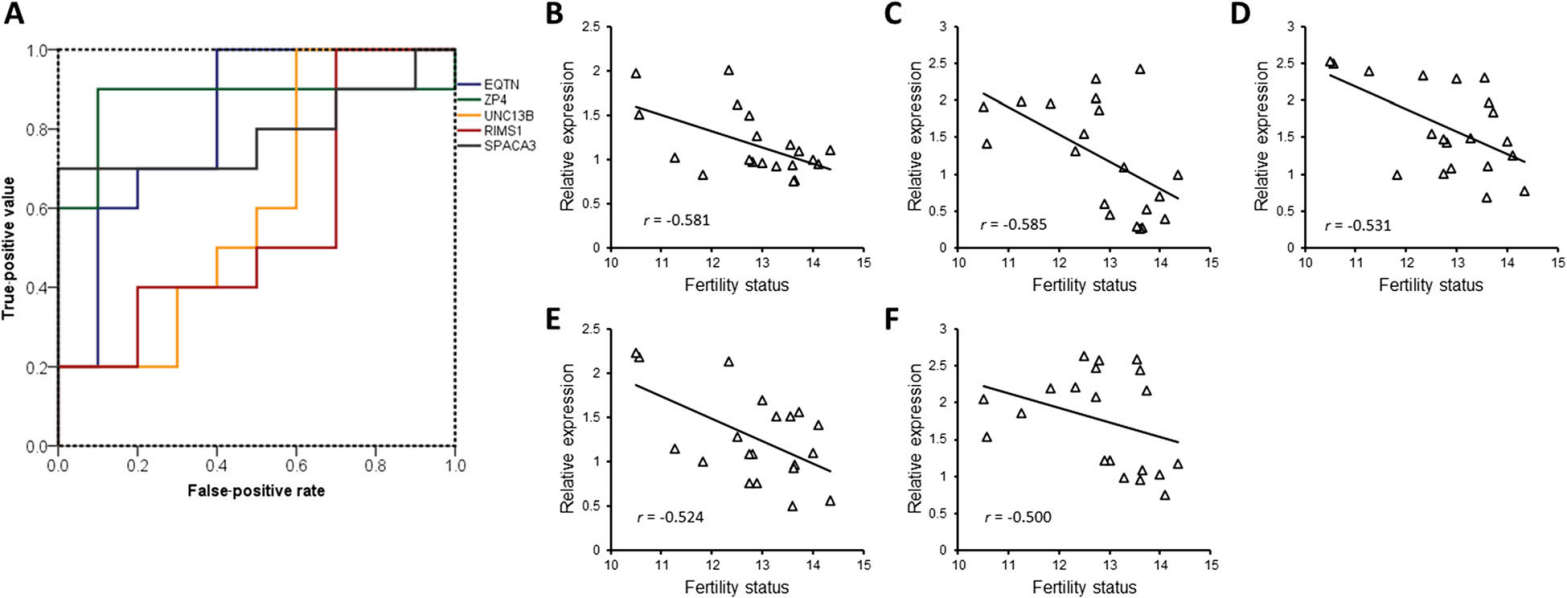
Assessment of single fertility markers. **A** The receiver operating characteristic curve of *EQTN, ZP4, UNC13B, RIMS1*, and *SPACA3* for single marker evaluation. Linear regression of (**B**) *EQTN*, (**C**) *ZP4*, (**D**) *UNC13B*, (**E**) *RIMS1*, and (**F**) *SPACA3* and fertility status of 20 randomly selected pigs. *r*, Pearson coefficient; *P* < 0.05

The expression of genes encoding *ZP4, RIMS1, RAB3A, UNC13B, SYT6, EQTN, IZUMO1, SPACA3, CD9*, and *IZUMO1R* were screened in 20 randomly selected boars. The fertility of boars was significantly correlated with the expression of *EQTN*, *ZP4*, *UNC13B*, *RIMS1*, and *SPACA3* (Fig. 2B–F; *P* < 0.05). The expression pattern of the genes encoding these five proteins in 20 randomly selected boars was the same as the expression pattern of the high- and low-fertility groups. The male fertility prediction value was assessed with ROC curve analysis [41]. The area under the curve ranged from 0.6 to 0.9, and overall accuracy (OA) ranged from 45% to 90% (Fig. 2A and Table 2). *ZP4* showed the highest values of sensitivity (SN), specificity (SP), negative predictive value (NPV), positive predictive value (PPV), and OA, all of which were 90% (Table 2). The OA of *UNC13B* and *RIMS1* were comparatively low (Table 2).

**Table 2:** Male fertility prediction value of single markers

Gene	Cut-off value	AUC	Sensitivity, %	Specificity, %	NPV, %	PPV, %	OA, %
*EQTN*	1.0	0.8	70.0	70.0	70.0	70.0	70.0
*ZP4*	1.2	0.9	90.0	90.0	90.0	90.0	90.0
*UNC13B*	1.5	0.6	60.0	50.0	55.6	54.5	55.0
*RIMS1*	1.1	0.6	50.0	40.0	44.4	45.5	45.0
*SPACA3*	2.0	0.8	70.0	70.0	70.0	70.0	70.0

*AUC* Area under the curve. Sensitivity is the percentage of pigs showing true-positive results when tested with mRNA expression. Specificity is the percentage of pigs showing true-negative results. The positive predictive value (PPV) is the percentage of pigs that tested as positive and simultaneously showed true-positive litter size. The negative predictive value (NPV) is the percentage of pigs that tested as negative and simultaneously showed true-negative litter size. *OA* overall accuracy

### Assessment and optimization of multiple fertility marker model

**Fig. 3:**
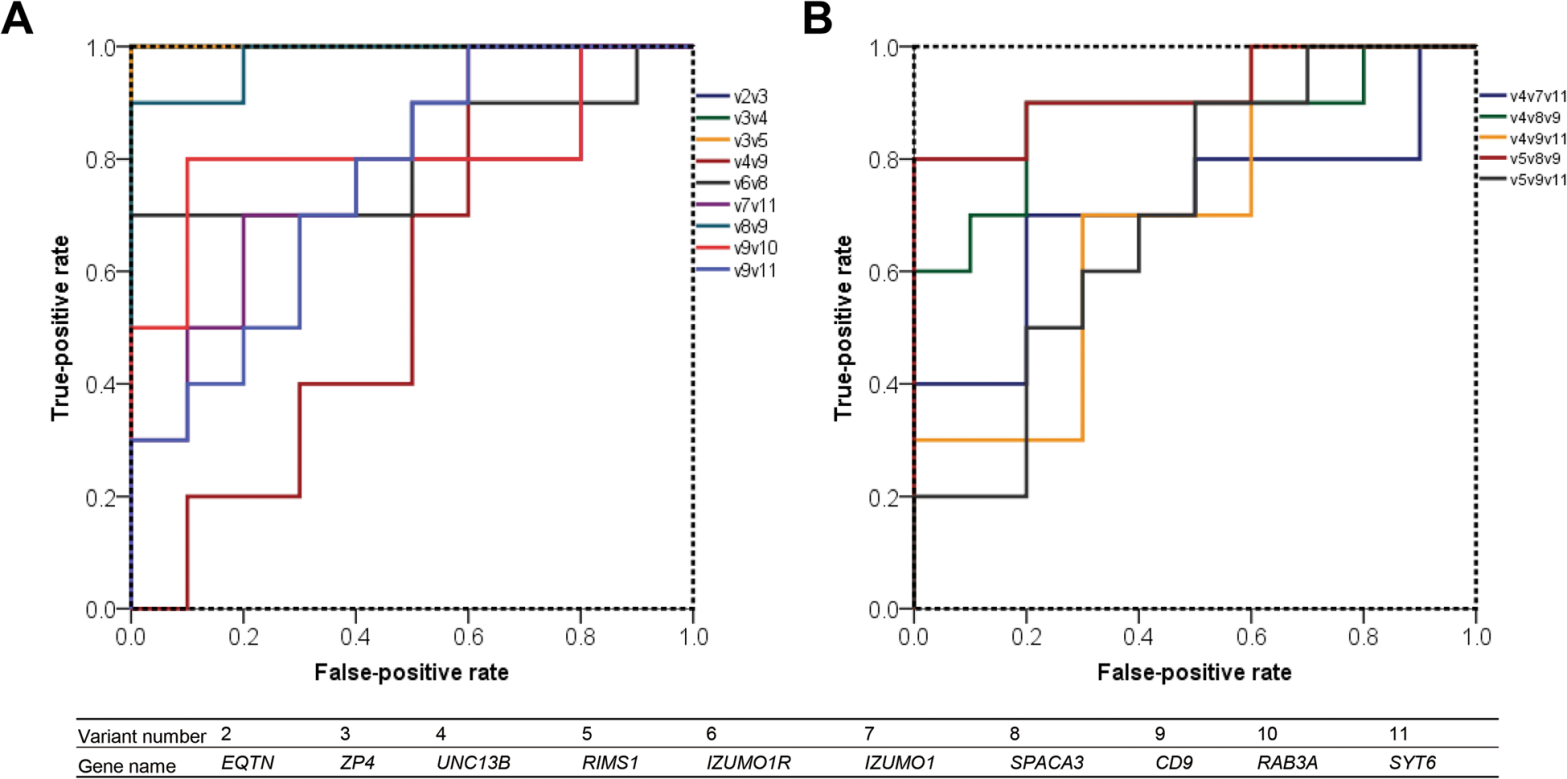
Assessment of multiple fertility markers. **A** The receiver operating characteristic curve of the predictive ability of the two-gene marker models for male fertility prediction. **B** The receiver operating characteristic curve of the predictive ability of the three-gene marker models for male fertility prediction

**Fig. 4:**
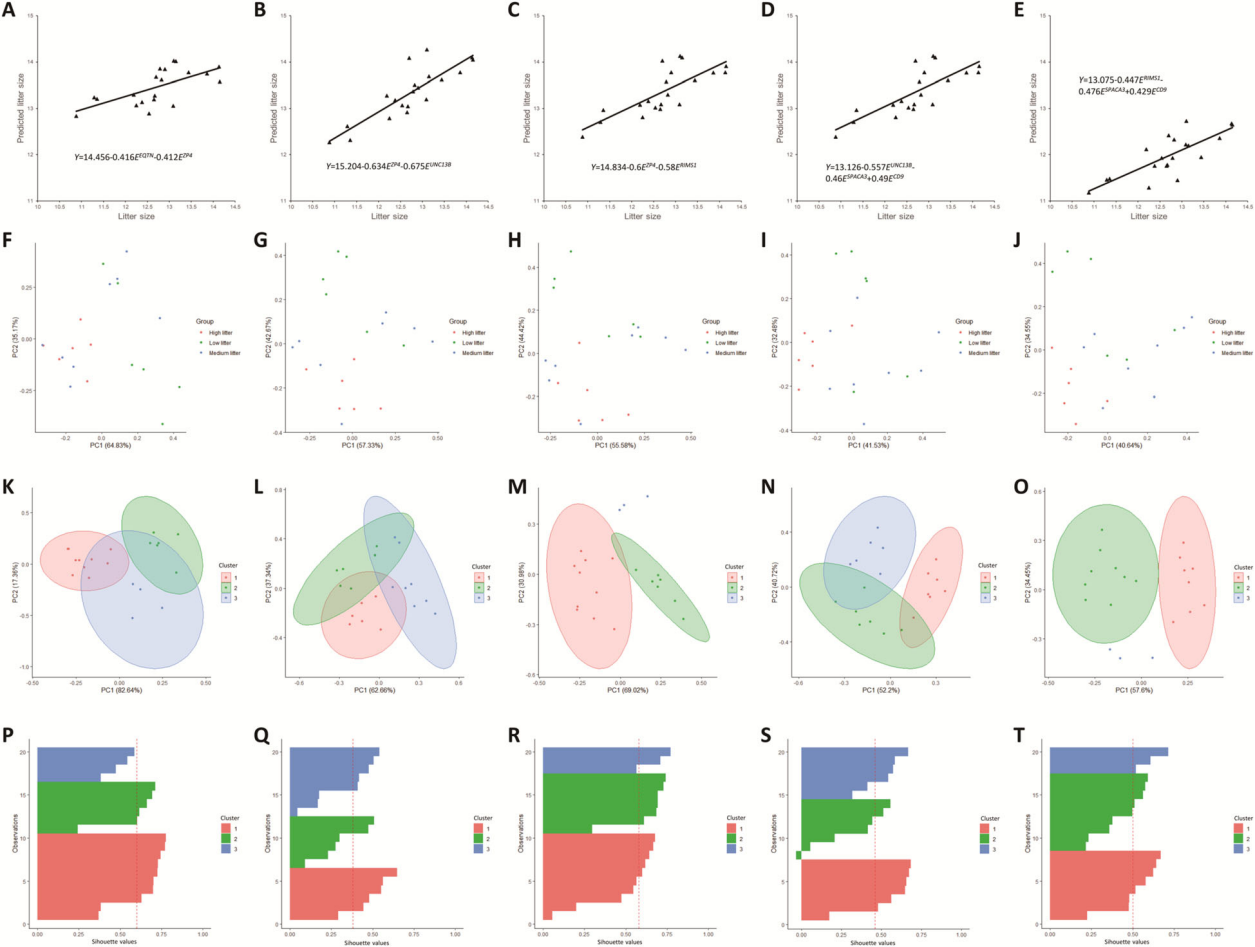
Multiple marker evaluation. Scatter plots of (**A**) *EQTN-ZP4*, (**B**) *ZP4-UNC13B*, (**C**) *ZP4- RIMS1*, (**D**) *UNC13B-SPACA3-CD9*, and (**E**) *RIMS1-SPACA3-CD9* prediction models (*E* = relative expression level of genes). Principal component analysis score plots of (**F**) *EQTN-ZP4*, (**G**) *ZP4-UNC13B*, (**H**) *ZP4- RIMS1*, (**I**) *UNC13B-SPACA3-CD9*, and (**J**) *RIMS1-SPACA3-CD9* prediction models to identify the high-, medium-, and low-fertility groups. *K*-medoids clustering plots of (**K**) *EQTN-ZP4*, (**L**) *ZP4-UNC13B*, (**M**) *ZP4- RIMS1*, (**N**) *UNC13B-SPACA3-CD9*, and (**O**) *RIMS1-SPACA3-CD9* prediction models to separate them into the high-, medium-, and low-fertility groups. Silhouette plots of (**P**) *EQTN-ZP4*, (**Q**) *ZP4-UNC13B*, (**R**) *ZP4- RIMS1*, (**S**) *UNC13B-SPACA3-CD9*, and (**T**) *RIMS1-SPACA3-CD9* prediction models to separate them into the high-, medium-, and low-fertility groups

**Fig. 5:**
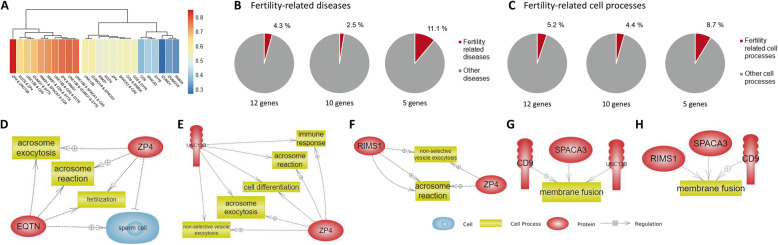
Heatmap and bioinformatics analysis of marker models. **A** Heatmap showing the coefficient values of the multiple-marker prediction models and single markers. **B** Pie chart denoting the percentage of fertility-related diseases associated with the different number of genes obtained by Pathway Studio. **C** Pie chart denoting the percentage of fertility-related cell processes associated with the different number of genes obtained by Pathway Studio. The common cell processes associated with genes in the (**D**) *EQTN-ZP4*, (**E**) *ZP4-UNC13B*, (**F**) *ZP4- RIMS1*, (**G**) *UNC13B-SPACA3-CD9*, and (**H**) *RIMS1-SPACA3-CD9* prediction models

To improve the clinical validity of the fertility marker candidate, we performed multiple regression tests using all possible marker combinations with *EQTN*, *ZP4*, *UNC13B*, *RIMS1*, and *SPACA3*. A total of 14 combinations were chosen to identify a significant marker model to predict male fertility (Table 3, Fig. 3). The combination of *EQTN*-*ZP4* (SN = 100%, SP = 100%, NPV = 100%, PPV = 100%, OA = 100%), *ZP4*-*UNC13B* (SN = 100%, SP = 100%, NPV = 100%, PPV = 100%, OA = 100%), and *ZP4*- *RIMS1* (SN = 100%, SP = 100%, NPV = 100%, PPV = 100%, OA = 100%) showed outstanding predictive value (Table 3, Fig. 3A; *P* < 0.05). The combinations of *UNC13B*-*SPACA3*-*CD9* (SN = 80%, SP = 80%, NPV = 80%, PPV = 80%, OA = 80%) and *RIMS1*-*SPACA3*-*CD9* (SN = 80%, SP = 80%, NPV = 80%, PPV = 80%, OA = 80%) showed the highest predictive values among three-marker predictive models (Table 3, Fig. 3B; *P* < 0.05). The multiple regression model was established (Fig. 4A–E). In the correlation heatmap, the multiple-marker models were more clustered and showed higher correlation value than the single-marker models (Fig. 5A, *P* < 0.05). To identify the predictive model most efficient in diagnosing male fertility in the high-, medium-, and low-fertility groups, PCA, k-medoids clustering, and silhouette plotting were employed. The average litter size of high-, medium-, and low-fertility groups after clustering with each prediction model is shown in Additional file 1: Table S3. The PCA plot showed that the *EQTN*-*ZP4* model is the most efficient in diagnosing the high-fertility group (Fig. 4F) compared to other multiple-marker models (Fig. 4G–J). Moreover, in clustering analysis, the *EQTN*-*ZP4* model was efficient in clustering the high-fertility group (Fig. 4F, K, and P) compared to other multiple-marker models (Fig. 4G–J, L–O, and Q–T). Compared to *EQTN*-*ZP4*, the *ZP4*-*RIMS1* and *RIMS1*-*SPACA3*-*CD9* models were more effective in clustering the low-fertility group by excluding the high- and medium-fertility groups (Fig. 4G, J, L, O, Q, and T).

**Table 3:** Male fertility prediction value of multiple marker models

Prediction model	AUC	Sensitivity, %	Specificity, %	NPV, %	PPV, %	OA, %
*EQTN-ZP4*	1.0	100.0	100.0	100.0	100.0	100.0
*ZP4-UNC13B*	1.0	100.0	100.0	100.0	100.0	100.0
*ZP4-RIMS1*	1.0	100.0	100.0	100.0	100.0	100.0
*UNC13B-CD9*	0.6	50.0	50.0	50.0	50.0	50.0
*IZUMO1R-SPACA3*	0.8	70.0	60.0	66.7	63.6	65.0
*IZUMO1-SYT6*	0.7	70.0	60.0	66.7	63.6	65.0
*SPACA3-CD9*	1.0	90.0	80.0	88.9	81.8	85.0
*CD9-RAB3A*	0.8	80.0	80.0	80.0	80.0	80.0
*CD9-SYT6*	0.8	70.0	70.0	70.0	70.0	70.0
*UNC13B-IZUMO1-SYT6*	0.7	70.0	60.0	66.7	63.6	65.0
*UNC13B-SPACA3-CD9*	0.9	80.0	80.0	80.0	80.0	80.0
*UNC13B-CD9-SYT6*	0.7	70.0	70.0	70.0	70.0	70.0
*RIMS1-SPACA3-CD9*	0.9	80.0	80.0	80.0	80.0	80.0
*RIMS1-CD9-SYT6*	0.7	70.0	60.0	66.7	63.6	65.0

*AUC* Area under the curve. Sensitivity is the percentage of pigs showing true-positive results when tested with mRNA expression. Specificity is the percentage of pigs showing true-negative results. The positive predictive value (PPV) is the percentage of pigs that tested as positive and simultaneously has a true-positive litter size. The negative predictive value (NPV) is the percentage of pigs that tested as negative or simultaneously had a true-negative litter size. *OA* overall accuracy

### Marker-related diseases and cell processes

To understand the functional significance of the genes contained in male fertility predictive models, the Pathway Studio program (v 12.0, Elsevier, Amsterdam, NL) was used, and the following gene sets were applied to investigate the changing patterns of marker-related diseases and cell processes: all 12 genes related to “acrosomal vesicle exocytosis” and “sperm–egg fusion,” 10 differentially expressed genes in the high- and low-fertility groups, and 5 genes passed the validation trial. The percentage of fertility-related diseases and cell processes changed according to the validation level of each gene set (Fig. 5B and C). It was the highest when the five genes that passed validation trials were applied to Pathway Studio to find related diseases (11.1%; Fig. 5B) and cell processes (8.7%; Fig. 5C). In fertility-related diseases, terms like “infertility,” “germ cell neoplasm,” “male infertility,” and “male sterility” were contained in the program (Additional file 2: Table S4). For the fertility-related cell processes, the terms like “acrosome exocytosis,” “sperm cell function,” “spermatogenesis,” “sperm-oocyte fusion,” and “sperm cell adhesion” were found (Additional file 2: Table S4). Specifically, the genes part of the male fertility predictive model was analyzed to find the related cell processes. We found that acrosome reaction is involved in all the two-gene male fertility predictive models (Fig. 5D–F). We also found that the genes in the *UNC13B*-*SPACA3*-*CD9* and *RIMS1*-*SPACA3*-*CD9* male fertility predictive models played a common role in membrane fusion (Fig. 5G and H).

## Discussion

To the best of our knowledge, no systemic empirical research exists addressing the question of how to translate basic research findings into new diagnoses in male infertility. This is the first translational study to predict male infertility using pigs with sperm RNA markers. In this study, we found that *EQTN*, *ZP4*, and *SPACA3* exhibited high accuracy as markers to evaluate male fertility. Combinations of *EQTN*-*ZP4*, *ZP4-UNC13B*, and *ZP4-RIMS1* showed the highest prediction value. The *EQTN*-*ZP4* model was efficient for clustering the high-fertility group and may be useful for selection of animal that has superior fertility. Compared to the *EQTN*-*ZP4* model, the *ZP4*-*RIMS1* model was more efficient in clustering the low-fertility group.

Evaluation of sperm quality is critical in the timely diagnosis and prevention of male infertility. Most diagnostic tools analyze the phenotype and morphology of spermatozoa. However, the clinical value of such current sperm evaluation methods is still debatable [3] because of the complexity of processes involved in achieving male fertility. Rodent models are widely used to understand the highly structured mechanism of male fertility [42, 43]. The short gestation period and well-established gene regulation technique are the powerful benefits of using a rodent model for investigating male fertility. However, it is hard to apply the findings from studies on rodents to humans or other large animals directly for the following reasons: (i) insufficient clinical/phenotypic information; (ii) lack of field/clinical trial; (iii) the high genetic diversity of human and other large animals; and (iv) the genetic differences between rodents and human/large animals. The pig model has high genetic and physiological similarities with humans [27, 44] and is a better candidate for reproductive research than rodent model. Recently, Yue et al. presented a pig germline genome engineering technique [45]. Moreover, huge and delicate fertility data acquired from AI help to get qualified field data. These factors make pigs more accessible in studying male fertility. Therefore, we selected the pig as a translational animal model to determine a prominent marker to evaluate male fertility.

Large-scale transcriptomic analysis in many mammalian species has revealed fertility-related differential gene expressions [20–24]. Although the huge dataset available is valuable for future male fertility studies, the clinical application of differentially expressed genes as markers is uncertain. Recently, our previous studies found fertility markers to predict male fertility [46–48]. However, because of the polygenicity of male fertility, a single marker is not enough to predict male fertility [49, 50]. Thus, we used a multiple-marker approach to establish an optimum male fertility prediction model. We made the following efforts: (i) listed the genes related to two critical biological events that determine fertilization success; (ii) found a single marker to predict male fertility by analyzing field data; (iii) tested combinations of single markers to establish multiple-marker prediction models, and (iv) evaluated the multiple-marker prediction models.

The marker genes *ZP4, EQTN*, and *SPACA3* showed high accuracy in male fertility prediction. ZP4 is known to play a critical role in the taxon-specific binding of the spermatozoa to the oocyte [51] and induce the acrosomal reaction in human spermatozoa in a dose-dependent manner in humans [52]. Although the function of the ZP4 protein in spermatozoa is unknown, its mRNA expression is found in rodents and humans [53]. Evaluation from an evolutionary perspective can help us understand how *ZP4* mRNA expression is associated with fertilization [54]. In *Arabidopsis*, short suspensor transcripts are expressed during male gametogenesis and conveyed to female gamete for zygotic translation. Paternally derived *ZP4* transcripts might affect fertilization by aiding in detecting transcripts in the zygote.

EQTN is expressed in the equatorial segment of spermatozoa and induces an acrosome reaction to help sperm–oocyte fusion [55–57]. EQTN protein was differentially expressed according to the fertility status of the pig [4]. SPACA3 protein is expressed in cow [58] and human spermatozoa [59], and it acts by interacting with other sperm proteins to facilitate fertilization [60]. In spermatozoa, mRNA-protein interaction is still unclear. Therefore, it is suggested that sperm mRNAs have roles separate from the functional roles of sperm proteins. In another view, a study by Gur and Breitbart suggested the presence of mitochondrial translation in spermatozoa [61] and provided insights into the physiological roles of *EQTN* and *SPACA3* transcripts in spermatozoa. mRNA levels cannot directly be correlated with protein expression as post-transcriptional processes are crucial in the final synthesis of the native protein [62]. Therefore, *EQTN* and *SPACA3* transcripts may be associated with male fertility through the regulation of complex processes including acrosome reaction, sperm–oocyte fusion, fertilization, and beyond. Although, the mRNA expression of *EQTN* and *SPACA3* are prominent markers for determining male fertility, the elucidation of the functional roles of mRNA in sperm warrants further research.

After identifying single fertility markers, we conducted multiple regression tests with all possible gene combinations to establish an optimum prediction model. Multiple markers for the diagnosis of various diseases, such as cancer and neurological diseases have been developed [63, 64]. We established the *EQTN*-*ZP4*, *ZP4*-*UNC13B*, and *ZP4*- *RIMS1* prediction models that showed 100% accuracy in the prediction of the fertility status in the validation trial data. All genes in the three-gene prediction models were related to acrosome reaction. This indicates the importance of acrosome reaction in fertilization success. Interestingly, *ZP4* and *UNC13B* showed “immune response” and “cell differentiation” as related cell processes. Thus, *ZP4* and *UNC13B* might be potential targets for future studies on unraveling the connection between immune response and fertility.

For further evaluation of the multiple-marker prediction models, PCA and clustering were conducted. PCA is a powerful tool to combine multiple factors that affect the main variable and has been used to identify biomarkers of various diseases [65–68]. In the livestock industry, identifying animals with superior genetics is crucial for increasing productivity. Therefore, the *EQTN*-*ZP4* model will be useful in this field because of its efficiency in clustering high-fertility groups. Although we only focused on the reproductive traits of pigs, other traits such as immune resistance, heat stress resistance, and growth rate could be improved by adopting the same approach. Compared to the *EQTN*-*ZP4* model, the *ZP4*- *RIMS1* and *RIMS1*-*SPACA3*-*CD9* models were more efficient in clustering the low-fertility group by excluding the high- and medium-fertility groups. Therefore, we suggest that these models may be useful in biomedical studies to detect male infertility/sterility.

## Conclusions

In conclusion, our study established an efficient male fertility prediction model using the pig model and multiple gene combinations. These genes were involved in biological events critical to male fertility. The *ZP4* gene is highly predictive of male fertility. *ZP4* in combination with *EQTN* has a higher predictive value and may be useful in the livestock industry. The *EQTN*-*ZP4* male fertility prediction model demonstrated outstanding predictive value in detecting the low-fertility group. Therefore, we postulate that this pig as a translational model and biomarker combination can be utilized to diagnose male infertility/sterility. However, as our study was conducted in a single pig stud, validation of our proposed prediction model requires further investigation and trans-species evaluation.

## Supplementary Information

**Additional file 1: Table S1.** Primers designed for RT-qPCR. **Table S2.** Sperm motility, motion kinematics, and capacitation status of 20 randomly selected boar spermatozoa. **Table S3.** Average litter size of high-, medium-, and low-fertility groups after clustering.

**Additional file 2: Table S4.** All fertility-related diseases and cell processes related with markers.

## Data Availability

The datasets from the current study are available from the corresponding author on reasonable request.

## Abbreviations

AI: Artificial insemination
ALH: Mean amplitude of head lateral displacement
BCF: Beat cross frequency
cDNA: Complementary DNA
CTC: Chlortetracycline
EQTN: Equatorin
HYP: Hyperactivation
IZUMO1: Izumo sperm–egg fusion protein 1
IZUMO1R: IZUMO1 receptor
LIN: Linearity
lncRNA: Long non-coding RNA
LYZL6: Lysozyme like protein 6
miRNA: MicroRNA
mRNA: Messenger RNA
NPV: Negative predictive value
OA: Overall accuracy
PCA: Principal component analysis
piRNA: Piwi-interacting RNA
PPV: Positive predictive value
qPCR: Quantitative polymerase chain reaction
RAB3A: Ras-related protein Rab-3A
RIMS1: Regulating synaptic membrane exocytosis protein 1
ROC: Receiver operating characteristic
siRNA: Short interfering RNA
SN: Sensitivity
SP: Specificity
SPACA3: Sperm acrosome membrane-associated protein 3
SPATA46: Spermatogenesis-associated protein 46
SYT6: Synaptotagmin-6
tRNA: Transfer RNA
tsRNA: Transfer RNA-derived small RNA
UNC13B: Protein unc-13 homolog B
VAP: Average path velocity
VCL: Curvilinear velocity
VSL: Straight-line velocity
WOB: Wobble
ZP4: Zona pellucida sperm-binding protein 4
