# Supplementary material for: Establishment of a male fertility prediction model with sperm RNA markers in pigs as a translational animal model
Source: J Anim Sci Biotechnol. 2022 Jul 7;13:84. doi: 10.1186/s40104-022-00729-9 (PMC9261079; doi:10.1186/s40104-022-00729-9)
Supplement: Supplementary file 1 — Additional file 1: Table S1. Primers designed for RT-qPCR. Table S2. Sperm motility, motion kinematics, and capacitation status of 20 randomly selected boar spermatozoa. Table S3. Average litter size of high-, medium-, and low-fertility groups after clustering. [file 40104_2022_729_MOESM1_ESM.docx]

Establishment of a male fertility prediction model with sperm RNA markers in pigs as a translational animal model

Won-Ki Pang, Shehreen Amjad, Do-Yeal Ryu, Elikanah Olusayo Adegoke, Md Saidur Rahman, Yoo-Jin Park, and Myung-Geol Pang*

Department of Animal Science & Technology and BET Research Institute, Chung-Ang University, Anseong, Gyeonggi-do, 17546, Republic of Korea

*Corresponding author:

Myung-Geol Pang

**Email:** mgpang@cau.ac.kr

Supplemental table

**Table S1. Primers designed for RT-qPCR.**

|  | Gene | Upper primer (5' - 3') | Tm (°C ) | Lower primer (5' - 3') | Tm (°C ) | Amplicon size (bp) |
| --- | --- | --- | --- | --- | --- | --- |
| Acrosomal vesicle exocytosis | ZP4 | ACC ATT GGC CTT TGT GAT GC | 64.6 | TCT TCC GAG TTG TAG CAG CA | 64.3 | 109 |
|  | RIMS1 | GGC CAT CAT CAA GAG CAC AAG | 64.5 | TTT CCA CCT GCT CCA ACT GT | 64.9 | 104 |
|  | RAB3A | TCC TCG GAC CAG AAC TTT GAC | 64.8 | TGC ATA GCG GAA GAG GAA GG | 64.8 | 89 |
|  | UNC13B | CCG GGA TGT CTT CAG TGT GA | 64.6 | GAT GGT AAT CTT GGC CGA CC | 63.5 | 105 |
|  | SYT6 | TGA ACC CCA CCT TCG ATG AG | 64.7 | GGA GAA GCG GTC AAA GTC GA | 65 | 100 |
| AVE and SEF | EQTN | AAA CCC TGC AAA TGA AGA CAA C | 63 | CTG CCA AAA TGA TGA CAA AAA G | 60 | 106 |
| Sperm-egg fusion | IZUMO1 | AAA GTC GTG GAG GAG CTG AG | 64.6 | ACC GCC TGC TTT ACC CTT TT | 65.6 | 100 |
|  | SPACA3 | GTC TGT CTG GCT TAC TTC GCA | 65.2 | ACT TCC GGC TGT TGA TCT GG | 65.2 | 102 |
|  | CD9 | CTG GAC TGC TGT GGT TTA ATG G | 64.4 | CTT CAT TGG GAG GCT TGA GAG T | 65.2 | 89 |
|  | LYZL6 | ACA GAC GGC AGC TTT GAC TA | 64.5 | ATG TTC TCC GTG TGA CTC CG | 64.8 | 82 |
|  | SPATA46 | TGG AGA CCT ACA GCT TCC CA | 65.5 | GGA GGA AGA GGA CGA GCA GA | 65.9 | 100 |
|  | IZUMO1R | TTC CCG CAT TAC TTC CCC AC | 65.3 | GGC TCG AAC CAC TTT TGC AG | 64.9 | 115 |
| Reference gene | GAPDH | AAG AGC ACG CGA GGA GGA G | 66.8 | GGG GTC TGG GAT GGA AAC T | 64.4 | 109 |
|  |  |  |  |  |  |  |

AVE: Acrosomal vesicle exocytosis; SEF: Sperm-egg fusion; All primers were designed genes from pig reference genome Sscrofa11.1 Primary Assembly. Gene accession number: ZP4 (ENSSSCG00000010141); RIMS1 (ENSSSCG00000004280); RAB3A (ENSSSCG00000013897); UNC13B (ENSSSCG00000005305); SYT6 (ENSSSCG00000006758); EQTN (ENSSSCG00000005121); IZUMO1 (ENSSSCG00000003142); SPACA3 (ENSSSCG00000017726); CD9 (ENSSSCG00000022230); LYZL6 (ENSSSCG00000017321); SPESP1 (ENSSSCG00000032244); SPATA46 (ENSSSCG00000006343); IZUMO1R (ENSSSCG00000014952); and GAPDH (ENSSSCG00000000694).

**Table S2. Sperm motility, motion kinematics, and capacitation status of 20 randomly selected boar spermatozoa.**

| Litter size | MOT, % | HYP, % | VCL, μm/s | VSL, μm/s | VAP, μm/s | LIN, % | BCF, Hz | WOB, % | ALH, μm | AR, % | F, % | B, % |
| --- | --- | --- | --- | --- | --- | --- | --- | --- | --- | --- | --- | --- |
| 14.4 | 89.4 | 25.9 | 164.8 | 75.8 | 85.1 | 46.0 | 10.9 | 51.6 | 7.2 | 2.0 | 80.3 | 17.7 |
| 14.1 | 94.2 | 22.6 | 160.5 | 60.8 | 78.0 | 37.9 | 11.7 | 48.6 | 7.0 | 2.4 | 84.4 | 13.2 |
| 14.0 | 83.4 | 18.4 | 147.2 | 62.3 | 74.1 | 42.3 | 11.7 | 50.3 | 6.4 | 1.9 | 92.2 | 5.9 |
| 13.7 | 72.9 | 9.5 | 138.7 | 74.4 | 79.7 | 53.6 | 12.2 | 57.5 | 6.3 | 1.2 | 89.3 | 9.6 |
| 13.6 | 94.9 | 36.6 | 181.9 | 75.3 | 91.2 | 41.4 | 10.5 | 50.2 | 7.9 | 1.1 | 88.9 | 10.0 |
| 13.6 | 85.6 | 13.9 | 138.8 | 59.3 | 70.9 | 42.7 | 11.5 | 51.1 | 6.1 | 0.0 | 92.9 | 7.1 |
| 13.6 | 61.8 | 5.1 | 109.8 | 53.1 | 60.6 | 48.2 | 13.0 | 55.1 | 4.9 | 0.0 | 93.9 | 6.1 |
| 13.5 | 74.3 | 8.7 | 119.7 | 49.1 | 60.3 | 41.0 | 13.0 | 50.4 | 5.3 | 0.4 | 95.2 | 4.3 |
| 13.3 | 71.5 | 7.2 | 136.6 | 80.4 | 82.5 | 58.8 | 12.3 | 60.4 | 6.3 | 0.3 | 91.3 | 8.4 |
| 13.0 | 85.6 | 20.8 | 153.3 | 66.0 | 78.8 | 43.0 | 11.5 | 51.0 | 6.6 | 0.0 | 93.1 | 6.9 |
| 12.9 | 74.2 | 12.3 | 126.8 | 49.7 | 61.3 | 39.2 | 12.6 | 48.3 | 5.5 | 0.9 | 87.7 | 11.5 |
| 12.8 | 86.2 | 21.0 | 149.9 | 58.3 | 72.4 | 38.9 | 11.7 | 48.3 | 6.5 | 2.7 | 64.1 | 33.2 |
| 12.7 | 74.3 | 11.3 | 130.8 | 52.1 | 63.5 | 39.8 | 12.2 | 48.5 | 5.7 | 3.9 | 83.8 | 12.3 |
| 12.7 | 62.3 | 5.0 | 110.2 | 54.0 | 61.0 | 50.8 | 20.0 | 59.1 | 4.9 | 7.9 | 74.9 | 17.2 |
| 12.5 | 79.0 | 13.2 | 131.6 | 48.4 | 62.2 | 36.8 | 12.4 | 47.3 | 5.8 | 9.2 | 83.8 | 7.0 |
| 12.3 | 78.4 | 15.5 | 136.8 | 53.6 | 64.1 | 39.2 | 11.8 | 46.9 | 5.8 | 0.0 | 97.6 | 2.4 |
| 11.8 | 80.2 | 13.1 | 133.4 | 55.3 | 67.6 | 41.4 | 11.9 | 50.7 | 5.8 | 0.3 | 96.8 | 2.9 |
| 11.3 | 64.7 | 9.8 | 118.0 | 48.8 | 58.7 | 41.4 | 13.0 | 49.8 | 5.2 | 10.5 | 70.2 | 19.4 |
| 10.6 | 92.6 | 27.3 | 166.0 | 59.9 | 77.1 | 36.1 | 11.7 | 46.4 | 7.1 | 1.6 | 88.6 | 9.8 |
| 10.5 | 76.4 | 8.7 | 120.7 | 49.1 | 60.3 | 41.0 | 13.0 | 50.4 | 5.3 | 0.1 | 91.8 | 8.1 |

MOT = motility; HYP = hyperactivation; VCL = curvilinear velocity; VSL = straight line velocity; VAP = average path velocity; LIN = linearity; BCF = beat cross frequency; WOB = wobble; ALH = mean amplitude of head lateral displacement; AR = acrosome reacted; F = live non-capacitated; B = live capacitated.

**Table S3. Average litter size of high-, medium-, and low-fertility groups after clustering.**

|  | High-fertility |  | Medium fertility |  | Low-fertility |  |
| --- | --- | --- | --- | --- | --- | --- |
| Marker combination | PCA analysis | *k-mddoid* clustering and silhouette plotting | PCA analysis | *k-mddoid* clustering and silhouette plotting | PCA analysis | *k-mddoid* clustering and silhouette plotting |
| *EQTN-ZP4* | 13.47 | 13.38 | 12.90 | 13.25 | 11.72 | 12.13 |
| *ZP4-UNC13B* | 13.47 | 13.34 | 12.90 | 12.88 | 11.72 | 11.72 |
| *ZP4-RIMS1* | 13.47 | 13.61 | 12.90 | 13.17 | 11.72 | 12.13 |
| *UNC13B-SPACA3-CD9* | 13.47 | 13.47 | 12.90 | 12.87 | 11.72 | 11.92 |
| *RIMS1-SPACA3-CD9* | 13.47 | 13.61 | 12.90 | 13.06 | 11.72 | 12.00 |

PCA = Principal component analysis. Data are expressed as mean ± SEM.
